# Controlled burn: interconnections between energy-spilling pathways and metabolic signaling in bacteria

**DOI:** 10.1128/jb.00542-24

**Published:** 2025-03-31

**Authors:** Nicolaus Jakowec, Steven E. Finkel

**Affiliations:** 1 Molecular and Computational Biology Section, Department of Biological Sciences, University of Southern California 118558, Los Angeles, California, USA; University of Notre Dame, Notre Dame, Indiana, USA

**Keywords:** stationary phase, starvation, futile cycles, energy metabolism, intracellular signaling, overflow metabolism, metabolic signaling

## Abstract

Bacterial energy-spilling pathways—such as overflow metabolism and futile cycles—have been considered inefficient forms of metabolism that result from poor regulatory control or function as mechanisms to cope with excess energy. However, mounting evidence places these seemingly wasteful reactions at the fulcrum between metabolic signaling and stress adaptation in bacteria. Specifically, energy-spilling pathways may mediate the metabolic reprogramming observed when cells encounter growth-limiting constraints (i.e., nutrient limitation). Recent insights spotlight microbial metabolism as an intricate signaling network that coordinates physiological programming with energy and nutrient conditions. Such intracellular metabolic cross stalk is pivotal to survival in competitive, fluctuating environments that bacteria frequently encounter in nature. In light of this paradigm of metabolic signaling, energy-spilling pathways are increasingly recognized as regulatory strategies that enable metabolic rewiring in response to stress. Overflow metabolism or futile cycles may generate secondary metabolites with signaling properties, alter the flux of metabolic pathways and the rate of nutrient acquisition, or stimulate regulatory nodes to trigger specific metabolic programs in response to environmental challenges. Furthermore, the observation of such expensive pathways under laboratory conditions purported to be “energy limiting” may in fact suggest energy sufficiency, compelling us to rethink how we model energy limitation and starvation for bacteria.

## INTRODUCTION

Bacteria are among the most metabolically versatile and diverse groups of organisms on Earth, enduring, and even thriving, across an astonishing range of ecological niches and environmental extremes. Despite this variety and versatility, most bacteria are subjected to a “feast-famine” lifestyle, consisting of periods of starvation interspersed with variable nutrient or energy inputs (1–4). Under such challenges, bacteria must not only endure starvation, in which growth is limited due to lack of energy or essential nutrients, but also adapt quickly when resources become available (5, 6). In the laboratory under conditions of starvation, bacteria enter a growth-arrested state termed stationary phase, a condition of lowered yet still active metabolism that supports essential cellular processes (7). To prolong survival under these conditions, bacteria can deploy an arsenal of mechanisms, including decreasing protein translation (8), reducing cell size and increasing cell wall rigidity (9, 10), and activating heat shock proteins and antioxidant defense systems to guard against damaging stressors (11, 12), among many others (13, 14). Responding to energy and nutrient inputs requires environmental sensing combined with the priming of metabolic pathways, orchestrated by specific transporters and catabolic enzymes (15, 16). Accordingly, bacteria often respond with a complex survival regimen, balancing the metabolic austerity of starvation with the plasticity required for adapting to dynamic environments. This paradigm, which asserts cellular austerity as a hallmark of starvation physiology, has largely been derived from work under laboratory conditions that supposedly recapitulate the growth-limiting settings microbes encounter in nature. However, not only are metabolic inefficiencies pervasive under starvation conditions, but also the laboratory conditions themselves often fail to create energy or nutrient limitation to the extent purported. This leads to the possibility that the lack of growth in culture may not represent a starvation state as previously thought.

It is generally accepted that competing and surviving in resource-limited environments demand tight metabolic control coupled with maximal energetic efficiency. However, a growing body of evidence challenges the long-standing notion of maximized bioenergetic efficiency in bacteria. Metabolic pathways that inefficiently utilize substrates or expend energy seemingly wastefully—termed “energy-spilling” reactions (17)—may in fact be a key metabolic strategy in the survival toolset, enabling the nutrient sensing and physiological fine-tuning that underpins survival in stressful and ever-changing environments. These metabolic pathways may not be biochemical anomalies or wasteful activities but function as essential metabolic drivers and regulators to achieve adaptive homeostasis. This rationale for seemingly wasteful metabolic pathways, and a potential answer to why cells “burn” energy apparently superfluously, originates from the expanding framework of microbial metabolic signaling.

Microbial survival in unstable or nutrient-scarce environments relies on the ability to both monitor fluctuations of intracellular metabolites and enact appropriate physiological responses. Intracellular monitoring is achieved via metabolites’ allosteric modulation of key enzymes, coupled with the regulation of specific transcription factors (18). In some cases, the concentration of the signal metabolite correlates linearly with the rate of the anabolic and catabolic pathways. Through this relationship, the rate or flux of a metabolic reaction is measured by the metabolite-protein interaction, conveying dynamic perturbations in metabolite levels and functioning as a signal that integrates the status of a variety of metabolic inputs. This “flux sensing” represents a critical form of feedback directly from metabolism onto cell signaling and transcription modulation (19, 20). In this model of regulatory control, the information conveyed by metabolic flux could be as significant as the metabolite’s bioenergetic or biosynthetic roles. The cell may even prioritize some metabolic pathway’s signaling capacity over its energy-harnessing potential; in other words, reactions that apparently “waste” or “spill” energy in fact could be harnessed for their regulatory functions.

Energy-spilling reactions have been long recognized as a pervasive phenomenon in microbial systems. It was long assumed that in batch culture experiments, biomass production (anabolism) and ATP generated from energy-yielding pathways (catabolism) were directly proportional. This may not be the case based on variations in ATP yield (21, 22). Studies in continuous culture systems demonstrated that variations in ATP yield can be attributed to the metabolic demands of non-growth processes, such as cellular maintenance activities and metabolic reprogramming. However, further studies demonstrated these maintenance processes alone could not address all yield variations (22, 23). The discrepancy between anabolism and catabolism in culture systems can be explained by energy-spilling reactions (17, 22, 24). Recent experiments have explored these discrepancies. Nanocalorimetry analysis measuring heat flow and metabolic activity shows disproportionately high heat production during the lag phase and stationary phase possibly attributed to energy-spilling reactions (25, 26).

Energy spilling represents the uncoupling between anabolism and catabolism (17), where carbon substrates are converted into waste by-products (overflow metabolism) (27–29) or ATP is dissipated due to the simultaneous activity of opposing reactions (futile cycles) (17, 30, 31). While possibly inefficient, such processes can serve as energy-balancing mechanisms under conditions of energy excess. For example, in the cyanobacterium *Synechocystis* sp., the synthesis of partially oxidized substrates during overflow metabolism can help regulate metabolic flux and contribute to energy balancing under high light and nitrogen-replete conditions (32). Recent studies have revealed insights into the function of energy-spilling reactions in competitive, multispecies environments or when cell growth is restricted due to limitation by energetic and nutrient sources (33–35).

The induction of energy-inefficient or energy-wasting pathways can function as a pivotal metabolic trade-off in cellular adaptation to challenging environments. In such scenarios, the signaling, detoxification, and pathway-bypassing roles of metabolites can supersede their theoretical maximal energy-furnishing capacity. Futile cycles, for example, can function as metabolic sinks to mitigate the buildup of toxic metabolites (36, 37), while overflow metabolism can generate metabolic intermediates that modulate transcriptional signaling or the activities of key enzymes (38, 39). The utility of apparent energy inefficiencies in microbial survival highlights that metabolic trade-offs contour the fitness landscape and spotlights metabolism’s elaborate and influential role in signaling and adaptive homeostasis. The purpose of this review is to reexamine energy-spilling pathways as vital mechanisms of metabolic adaptation in bacterial response to challenging and dynamic environments.

## ENOUGH ENERGY TO STARVE: MECHANISMS OF METABOLIC ADAPTATION TO THE STATIONARY PHASE

For microorganisms, metabolism embodies the biochemical interface between the genome and the environment. In addition to furnishing the cell with biomolecular building blocks and energy, microbial metabolism comprises a biochemical network interwoven with regulatory circuits to coordinate metabolic activities with extracellular contexts and the intracellular milieu (40). Allosteric and transcriptional regulations, mediated by protein-metabolite interactions, enable this metabolic fine-tuning and physiological reprogramming (41). Although genome-scale metabolic modeling and other high-throughput techniques have identified regulatory metabolites (18, 42), the extent of this form of genotype-phenotype interaction is not fully understood. This unfolding framework of metabolic signaling enriches our understanding of how bacteria harmonize cellular processes with environmental conditions.

**Fig 1:**
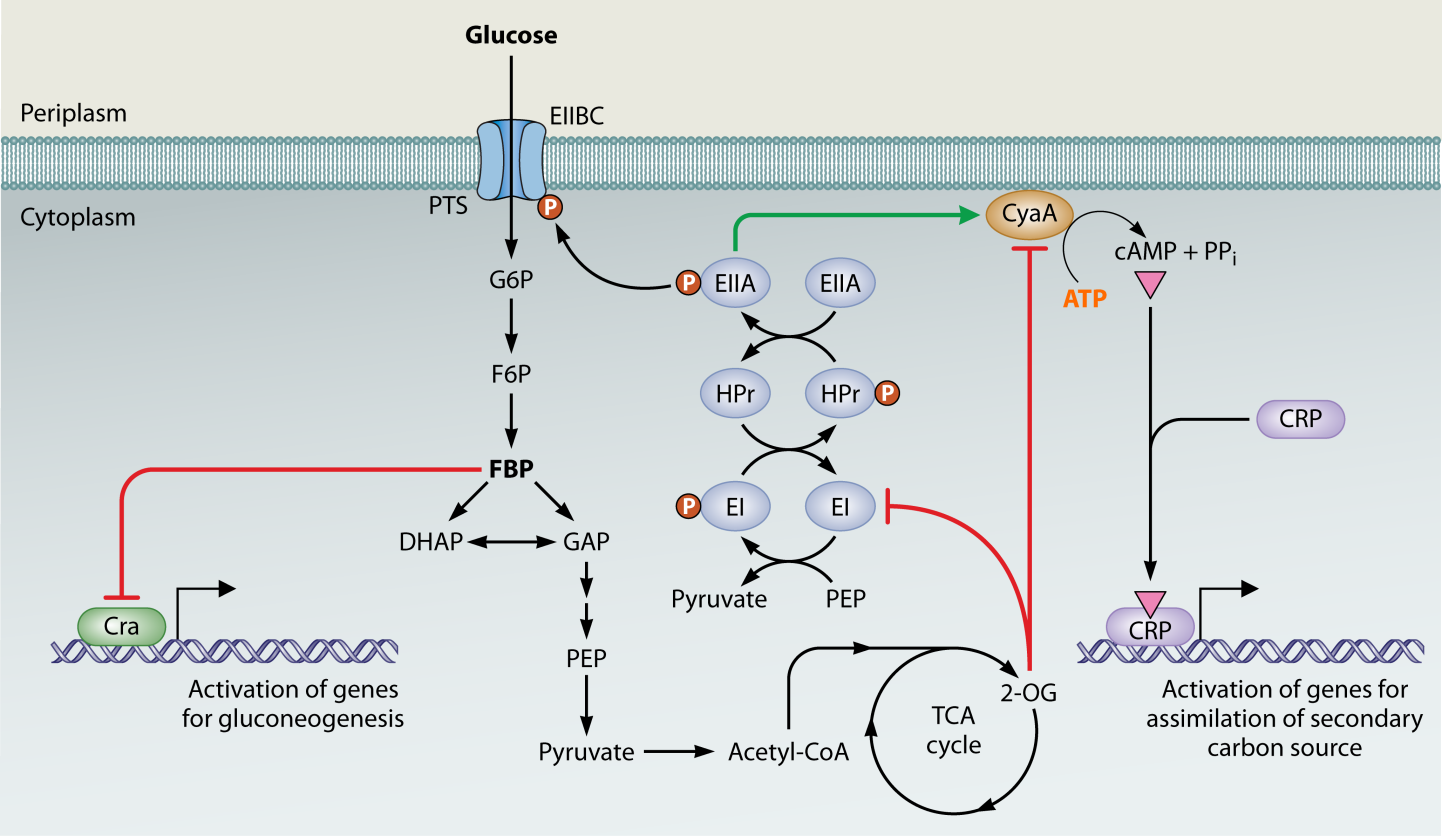
Flux sensing influences metabolic regulation via transcriptional and post-translational mechanisms in *E. coli*. Central carbon metabolism is embedded with several flux-sensing systems that translate metabolic flux into transcription factor activity, coupling carbon uptake with the expression of genes for carbon assimilation. For example, the metabolite fructose bisphosphate (FBP) is responsible for glycolytic flux-dependent regulation and inhibits the activity of the transcription factor Cra, which activates gluconeogenesis-related genes and inhibits genes encoding glycolytic enzymes. Glucose flux through the EIIBC component of the PTS affects the phosphorylation state of proteins in the phosphorylation cascade (EI, HPr, EIIA, and EIIB), which can interact with numerous non-PTS proteins, including adenylate cyclase (CyaA). When glucose flux is low, the phosphorylated form of EIIA activates adenylate cyclase, which synthesizes the secondary messenger cAMP from ATP. cAMP activates the global regulator CRP, which activates genes for assimilation of secondary carbon sources. Levels of the tricarboxylic acid (TCA) cycle intermediate 2-oxoglutarate (2-OG) increase under nitrogen limitation. By inhibiting EI of the phosphorylation cascade and CyaA, 2-OG blocks glucose uptake and downregulates genes for carbon utilization to coordinate carbon uptake with nitrogen availability.

Post-translational modifications (PTMs) provide rapid and reversible regulatory control in an ever-changing environment. Through enzyme modifications, such as acetylation or phosphorylation, small metabolites can modulate enzyme activities through allosteric or competitive mechanisms, allowing rapid physiological adaptations during nutrient shifts in source or abundance (43). Fifty-six percent of covalently modified proteins in *Escherichia coli* serve metabolic regulatory roles, indicating a vital role for PTMs in the global regulation of metabolism (44). While many PTMs remain to be identified, it is increasingly evident that key donor groups for PTMs originate from intermediates of central carbon metabolism. Through this interplay, several metabolites serve as indicators of the cell’s nutritional state and help coordinate carbon availability with appropriate enzyme activities. For example, the central carbon metabolite acetyl-CoA donates its acetyl moiety for the acetylation and inhibition of its synthesizing enzyme, acetyl-CoA synthetase, demonstrating a form of feedback inhibition (45, 46). Further, the glycolytic intermediate and high-energy phosphoryl donor phosphoenolpyruvate (PEP) can donate phosphate groups to modify protein conformation and regulate enzymatic activity. For example, the enzyme I (EI) subunit of the phosphotransferase system (PTS), essential for intracellular transport of a range of sugars, transfers a phosphoryl group from PEP to histidine-phosphorylatable phosphocarrier protein (HPr), which can transfer its phosphoryl group to a sugar-specific permease complex (enzymes EIIA/EIIBC). In contrast, when levels of a preferred sugar, such as glucose, are low, HPr remains phosphorylated and acts as a crucial regulator of downstream carbon and energy metabolism (47, 48). Additionally, enzyme EIIA phosphorylation, which regulates cyclic AMP (cAMP) synthesis and thus the activity of the catabolite repression transcription factor CRP (cyclic AMP receptor protein), is not only sensitive to glucose levels but is also influenced by the PEP:pyruvate ratio (49). Therefore, PEP-dependent phosphorylation of HPr functions as a key link between carbon and energy availability and physiological adaptation (40) (Fig. 1).

In addition to PTMs, key intermediates of central carbon metabolism and various metabolites can allosterically interact with proteins to induce regulatory changes. ATP, a direct indicator of cellular energy abundance, modulates enzyme activity, often in proportion to the levels of ADP and AMP (40). Experimental evidence suggests that the intracellular ATP amount regulates glycolysis since introducing an artificial ‘ATP sink’ by overexpressing an ATP-dissipating ATPase in *E. coli* can increase glycolytic flux (50). ATP and AMP inhibit phosphorylation and dephosphorylation, respectively, of fructose-6-phosphate by the glycolytic enzyme fructose bisphosphatase (Fbp), leading to increased glycolysis under conditions of limited ATP (51, 52). Intermediates of central carbon metabolism also contribute to this feedback mechanism. For example, the tricarboxylic acid (TCA) cycle intermediate 2-oxoglutarate and other α-ketoacids are emerging as important regulatory metabolites that lie at the intersection of carbon and nitrogen pathways (40, 53). 2-Oxoglutarate (2-OG, also known as α-ketoglutarate) and other α-ketoacids, such as pyruvate and oxaloacetate, are the direct carbon precursors for the transamination reaction in amino acid biosynthesis. As the carbon skeletons for nitrogen assimilation reactions, 2-OG levels are sensitive to both carbon and nitrogen availability and reflect the balance between catabolic and anabolic metabolic pathways. Under nitrogen-limiting conditions, 2-OG concentrations increase, which inhibits adenylate cyclase activity and thereby reduces the synthesis of cAMP, an intracellular messenger that activates carbon catabolic pathways (54, 55). In *E. coli*, 2-OG also blocks glucose uptake by inhibiting the EI subunit of the PTS, further coordinating carbon utilization with nitrogen availability (53) (Fig. 1). In cyanobacteria, 2-OG acts as an allosteric effector of the global transcriptional regulator NtcA. Under conditions of reduced nitrogen availability, 2-OG concentrations are elevated. 2-OG binds to NtcA and increases its affinity for DNA-binding targets, resulting in the upregulation of many genes, including those for nitrogen assimilation (56, 57). 2-OG and other α-ketoacids are biosynthetic precursors, intermediates of central carbon metabolism, and can modulate global transcriptional regulator activity. They act as metabolite sensors coupling carbon catabolic processes with nitrogen-mediated biosynthetic potential.

Beyond detecting the real-time concentrations of intracellular nutrients, cells can monitor and control the rates of metabolic reactions by sensing metabolite concentrations that directly correlate with reaction fluxes. So-called flux-signaling metabolites, such as fructose-1,6-bisphosphate (FBP) and cAMP, allosterically bind to their target enzymes or regulatory transcription factors to create flux sensors and enable flux-dependent regulation. Since these flux-signaling metabolites integrate inputs from multiple substrates and intermediates, flux sensing may offer a cost-efficient and sensitive regulatory scheme that alleviates the burden of expressing many nutrient-specific sensors simultaneously (58, 59). The glycolytic intermediate FBP has been identified as a metabolite that uses both allosteric and transcriptional regulation to modulate glycolytic flux. FBP activates the downstream glycolytic enzymes, pyruvate kinase and PEP carboxylase, to balance lower glycolysis with upper glycolytic flux (20). Regardless of the glycolytic carbon source, FBP can act as an allosteric inhibitor of the transcription factor Cra, which represses glycolytic enzymes while activating genes involved in gluconeogenesis in *E. coli*. Cra activity decreases linearly with glycolytic flux, detected by altered FBP pools, such that the switch from glycolysis to gluconeogenesis correlates with a key signal of glycolytic substrate availability (60). *E. coli* also regulates metabolism by another flux-sensing mechanism through the intracellular messenger cAMP (61). cAMP, synthesized by the membrane-bound adenylate cyclase, is activated by phosphorylated EIIA, a component of the PTS. The PTS concomitantly transports and phosphorylates glucose. This glucose uptake system also functions as a key regulatory node for catabolite repression, reducing alternative nutrient uptake when glucose, the preferred carbon source, is available. When glucose flux through the PTS is low, EIIA is phosphorylated, leading to cAMP synthesis (62). cAMP binds and further activates the transcription factor CRP, a regulator of over 180 genes in *E. coli*, many of which are involved in the catabolism of secondary carbon sources (55, 63) (Fig. 1). Other PEP:carbohydrate phosphotransferase systems also participate in this form of cross talk. For example, the mannose-specific and trehalose-specific PTS permeases couple their respective carbohydrate phosphorylation with adenylate cyclase activity (64). Thus, cells can utilize flux-signaling metabolite concentrations, which correspond with external nutrient levels, as internal signals to rewire metabolism in fluctuating environments. This integrative, dynamic signaling adaptation reveals mechanisms by which cells transpose perturbations of nutrients and metabolite levels into regulatory decisions and system-level modifications for survival.

The mechanisms for metabolic coordination are critical to survival in dynamic environments, in which cells must compete for limited resources and remodel their physiology in response to nutrient and energy limitations. Paradoxically, cells must maintain active metabolism and generate energy to effectively starve since the entry, persistence, and emergence from periods of energy limitation and growth arrest are made possible by energy-dependent reprogramming. Adaptation to growth-limiting conditions requires energy-dependent processes, such as ongoing protein synthesis and enzyme activity, to achieve metabolic reorganization (65). This adaptive homeostatic reorganization entails maintaining energy supply and biosynthetic intermediates to sustain essential cellular activities and protect against potential stressors by storing nutrient reserves, rerouting metabolic pathways, and suppressing overall high-demand cellular expenditures. This supply of “maintenance energy,” defined as the minimum amount of energy to sustain essential cellular functions and prevent cell death, is vital to enduring periods of starvation and also allowing cells to return to normal growth phase when environmental conditions become more favorable, which is reflected by adequate metabolic sources (65, 66). During competition for limited resources, cells must reenter the growth phase rapidly upon nutrient availability and must integrate external nutrient signals and modulate cellular activities appropriately. During growth arrest, cells must sense fluctuations of intracellular pools and regulate energy and nutrient use and acquisition (66–68). How bacteria integrate these internal signals of energy and nutrient levels during starvation remains a gap in our knowledge. Energy-spilling pathways, previously considered as wasteful metabolic pathways, may be an unrecognized mechanism of metabolic reprogramming during growth arrest.

## FLIPPING THE SWITCH ON ENERGY-SPILLING PATHWAYS

Energy-spilling pathways, characterized by seemingly wasteful energy expenditure, are enigmatic phenomena pervading microbial systems thought to be steered by natural selection toward the goal of maximal energy efficiency (17). The partial oxidation and subsequent excretion of metabolites (overflow metabolism) and the simultaneous activity of opposing biochemical pathways, leading to net energy loss (futile cycles), illustrate two modes of energy-spilling pathways established in a diverse array of prokaryotes. While historically characterized as mechanisms to cope with energy or substrate surplus during growth (22), energy-spilling pathways are also affiliated with growth-arrested states (33–35). The presence of “wasteful” metabolic processes during energy-limiting conditions potentially hints at a trade-off between energy production and metabolic signaling. Alternatively, the observation of wasteful pathways, such as futile cycling, during “energy-limiting” conditions might suggest that such circumstances may not actually be so energy limiting. The coupling of cell metabolism with regulatory mechanisms indicates that cells may leverage the signaling properties of a pathway, despite having a short-term metabolic cost, to organize global metabolism and facilitate physiological reprogramming during stress, including limited metabolic resources. In support of this model, an emerging body of evidence indicates that these energy-spilling pathways serve as regulatory strategies that enable reprogramming during growth arrest, where the signaling activities of such pathways are prioritized over their energy-furnishing or biosynthetic functions (Table 1).

**TABLE 1:** Summary of bacterial energy-spilling pathways with regulatory functions^*a*^

Species/strain	Energy-spiling mechanism	Metabolic pathway	Regulatory function	References
*E. coli*	Overflow metabolism	Acetate overflow production	Global transcriptional and flux regulation of central carbon metabolism	(29)
*Synechocystis* sp. *PCC 6803*	Overflow metabolism	Pyruvate and 2-oxoglutarate production	Energy balancing under high light intensities	(32)
*E. coli*	Futile cycle	Trehalose periplasmic recycling	Modulation of cAMP-CRP signaling during the stationary phase	(34)
*Mycobacterium tuberculosis*	Futile cycle	Trehalose recycling	Promoting energy-efficient mycomembrane adaptation to nutrient limitation	(35)
*Staphylococcus aureus*	Overflow metabolism	CidC and AlsSD overflow pathways	Modulating cell death to achieve optimal biofilm biomass	(38)
*E. coli*	Overflow metabolism	Acetate overflow production	Regulating metabolic shift after glucose depletion	(69)
*Streptococcus mutans*	Overflow metabolism	Acetate and pyruvate overflow production	Inducing stationary phase reprogramming for uptake of organic acids	(70)
*Salmonella enterica*	Overflow metabolism	Methylglyoxal overflow production	Maintaining optimal aerobic respiration and mediating adaptive response to oxidative stress	(71)
*E. coli* and *Pseudomonas aeruginosa*	Futile cycle	Simulation of simultaneous activity of four irreversible glycolytic and gluconeogenic reactions (i.e., Fbp and PfkA interconverting F6P and FBP)	Priming central carbon metabolism for shifts between glycolysis and gluconeogenesis	(72)
*E. coli*	Futile cycle	ATP hydrolysis by soluble F_1_ part of F_0_F_1_-ATP synthase	Global transcriptional and glycolytic flux regulation to maintain energy homeostasis, including increased glycolytic flux	(73)

^*a*^ AlsSD, acetolactate synthase/decarboxylase; CidC, pyruvate oxidase; FBP, fructose-1,6-bisphosphatase.

## OVERFLOW METABOLISM: BRIMMING WITH POTENTIAL

During glycolysis, even when oxygen is abundant, cells may produce and secrete seemingly wasteful by-products, such as acetate. This and other forms of aerobic fermentation are termed overflow metabolism (74). The shift away from oxidative phosphorylation (OXPHOS) toward fermentation and substrate-level phosphorylation yields a lower net energy output in terms of ATP production. This apparent contradiction in metabolism may offer a beneficial strategy under restricted conditions, such as complex competitive environments with multiple bacterial strains and species (75). Overflow metabolism has been generally characterized as a response to energy and nutrient excess. Such conditions can interfere with the coupling of anabolism and catabolism, leading to cells activating fermentation pathways and side reactions to modulate glycolytic flux or to recycle cofactors, such as NADH. This hypothesized “energy buffer” model, where side reactions are activated in response to excess energy inputs and metabolic bottlenecks, has prevailed as the primary theory for the function of overflow metabolism in microorganisms (32, 59). By mitigating the buildup of glycolytic intermediates and facilitating the turnover of redox cofactors, overflow metabolism can increase metabolic flux and resource competitiveness in dynamic environments (76–78). In addition to these characterized functions, the growth inhibition of bystander species due to the organic acid products of fermentation reactions can provide a competitive advantage (79). For example, *E. coli* transposon mutants of genes encoding the TCA cycle and oxidative phosphorylation enzymes and complexes result in enhanced overflow metabolism and manifest a selective advantage against *Pseudomonas aeruginosa* when cocultured in high glucose concentrations (75). Furthermore, recent findings implicate overflow metabolites in gene regulation and stress responses (69, 80) (discussed below), supporting the advantageous utility of a once seemingly wasteful metabolic phenomenon.

Overflow metabolism is increasingly associated with growth-limiting states and not only with energy excess conditions. The physiological organization during the stationary phase reduces enzyme levels and limits membrane space, such that metabolic pathways can become bottlenecked even when their substrates are not plentiful (28, 33). Other constraints that can promote overflow metabolism over the more efficient respiration to generate ATP include the protein synthesis cost of respiration (TCA cycle enzymes) (28), limited oxygen consumption (29, 74), and membrane crowding (81, 82). Overflow metabolism thus may not simply be a hallmark of fast growth during periods of energy excess but also may be a physiological strategy to cope with the deleterious demands of growth-limiting environments. In addition, aerobic fermentation can mitigate sources of intracellular stress during growth stasis. Avoiding cellular respiration and OXPHOS reduces reactive oxidative species (ROS) levels, diminishing a key source for molecular damage (83). Furthermore, some overflow products, such as α-ketoacids like pyruvate, can effectively scavenge ROS, including hydrogen peroxide, through non-enzymatic oxidative decarboxylation (84). In addition to enhancing robustness under conditions of excess, overflow metabolism is an important metabolic strategy during stationary phase physiology. Thus, overflow metabolism during stress, coupled with the signaling properties of some organic acids, implicates a regulatory role of overflow despite its metabolic inefficiency.

**Fig 2:**
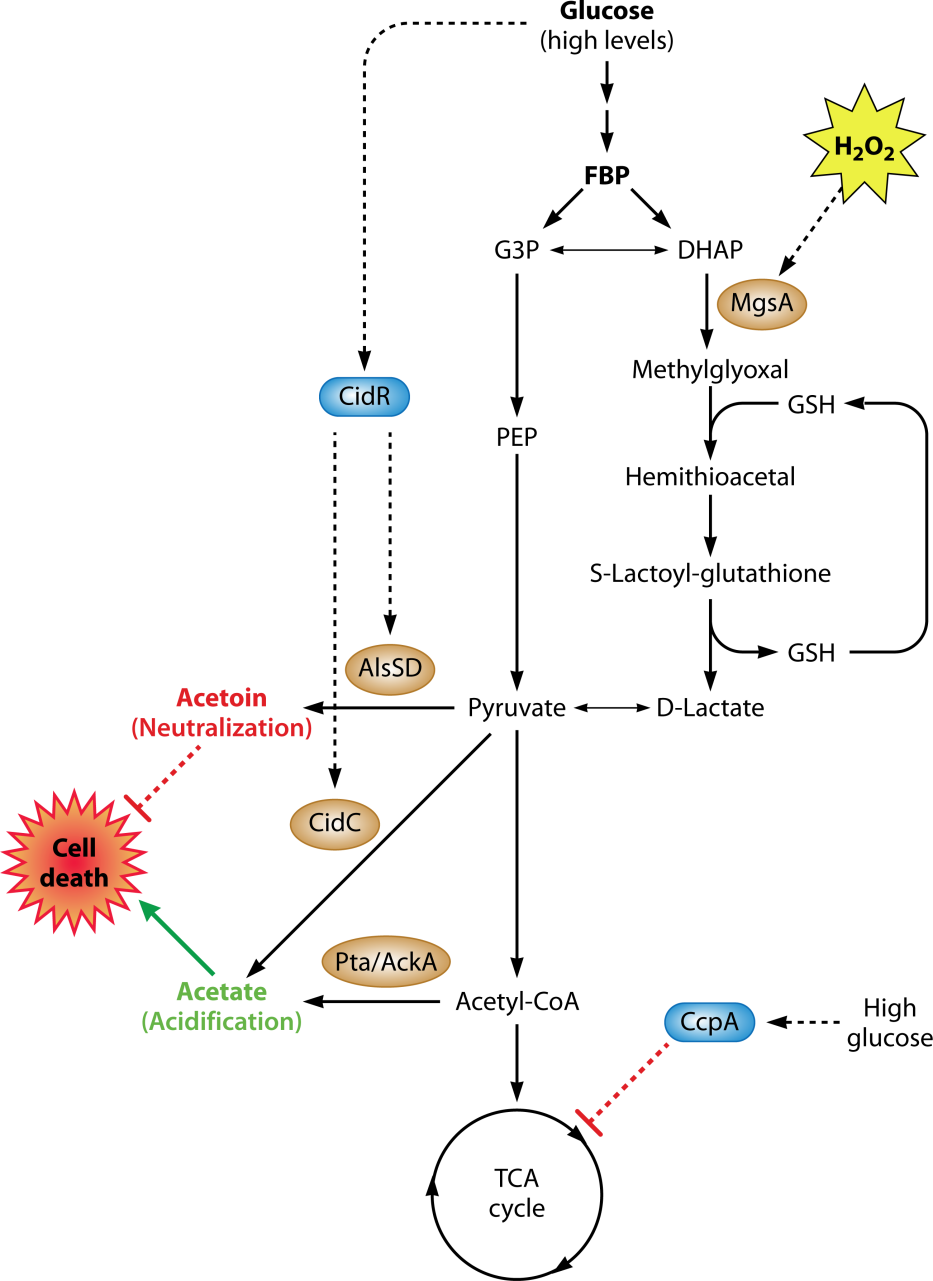
Carbon overflow pathways can modulate bacterial cell death and mediate metabolic adaptation during the stationary phase. *S. aureus* cultured in excess glucose undergoes CcpA-mediated catabolite repression, which inhibits the TCA cycle and increases activation of the transcriptional regulator CidR, which upregulates enzymes of overflow metabolism: acetolactate synthase/decarboxylase (AlsSD) and pyruvate oxidase (CidC). AlsSD and CidC convert pyruvate into the metabolites acetoin and acetate, respectively. While acetoin counters weak acid-mediated intracellular acidification of the cytoplasm, acetic acid-mediated intracellular acidification triggers cell death. The activities of both the AlsSD and CidC pathways modulate cell death at the population level, affecting the amount of cell death biomass important for achieving optimal biomass and structural integrity during biofilm development (38, 86). In *S. enterica*, peroxide stress induces expression of the methylglyoxal pathway—via upregulation of methylglyoxal synthase (*mgsA*)—an overflow metabolic pathway of glycolysis. This pathway enables increased glucose utilization necessary to satisfy the energetic, biosynthetic, and redox demands of oxidative stress resistance. In addition, methylglyoxal detoxification by its reaction with glutathione (GSH) consumes GSH, which is recycled from the S-lactoyl-glutathione as D-lactate is produced (71).

Recent findings suggest that by infusing the cell with key regulatory metabolites, overflow pathways can modulate stationary phase physiology and stress responses. In *Staphylococcus aureus*, for example, two carbon overflow pathways, the pyruvate oxidase (CidC) and the α-acetolactate synthase/decarboxylase (AlsSD) reactions, modulate programmed cell death (PCD) through their interaction with a transcriptional regulator, CidR (38) (Fig. 2). When *S. aureus* is cultured in excess glucose, CcpA-mediated catabolite repression leads to repression of the TCA cycle and a buildup of acetate in the medium, a by-product of glucose metabolism (85). High concentrations of acetate are growth inhibitory and, via generation of ROS and subsequent DNA damage, drive cell death during the stationary phase. However, acidic acetate also activates the expression of the CidR regulon, which controls genes encoding the CidC and AlsSD pathways, converting pyruvate into the fermentation products acetate and acetoin, respectively. Through CidC activity, this feedforward loop of acetate-induced overflow leads to toxic acetate levels, culminating in cell death in a subpopulation of cells. To prevent a disproportionate amount of cell death, the AlsSD counters CidC-mediated acetate accumulation by diverting carbon flux toward neutral by-product acetoin synthesis. Therefore, by adjusting acetate synthesis under glucose excess, CidC and AlsSD modulate acetate-mediated cell death. This cell death, regulated by overflow pathways, is necessary for optimal biofilm development and supporting the surviving population of *S. aureus* cells. By conferring surviving subpopulations with nutrients and biofilm-building matrix components, PCD promotes the structural and developmental integrity of the maturing biofilm (38, 86). A similar mechanism can also contribute to the pathogenesis of *S. aureus*, as demonstrated by enhanced bacterial colonization in a rabbit model of infective endocarditis (38).

In *Streptococcus mutans*, acetate and potassium (K^+^) coordinate the expression of *lrgAB*, encoding a pyruvate transporter during the stationary phase (70, 87). When cultivated in glucose-supplemented media, *S. mutans* secretes pyruvate as an overflow metabolite. After nutrients become limiting and *S. mutans* transitions to the stationary phase, cells initiate reuptake of excreted pyruvate through LgrAB. Transcripts for *lgrAB* are expressed during the exponential phase but then repressed during the stationary phase, even in the presence of external pyruvate. To alleviate transcript repression, acetate, released as an overflow product, together with K^+^, which can accumulate under stress conditions, activates the *lgrAB* promoter. Thus, acetate can function as an environmental cue for pyruvate availability since it is also a major by-product of pyruvate-H_2_O_2_ reactivity. Although the mode of acetate regulation is not fully understood, the mechanism may be indirect. It is postulated that excess acetate alters levels of acetyl phosphate, which phosphorylates and activates transcription factors, including LytT, responsible for promoting *lgrAB* transcription (70). Thus, overflow metabolism mediates a crucial metabolic shift during the stationary phase, enabling cells to adapt to alternative carbon sources, providing a metabolic advantage.

Another overflow metabolite implicated in stress responses is methylglyoxal, a highly reactive carbonyl compound and toxic by-product of elevated carbon flow through glycolysis. Methylglyoxal (MGO) synthesis is activated under conditions of glucose excess (88) as well as oxidative stress (71). Although a cytotoxic by-product of glycolysis, MGO can also function as a stress-signaling molecule at low concentrations, inducing adaptive responses to glycation and oxidative stress (89). MGO reacts with biomolecules such as DNA and proteins, forming a major precursor of advanced glycation end products, which can promote oxidative stress (90, 91). Activation of the MGO pathway can also support metabolic flexibility and help mitigate the cytotoxicity of ROS. In *Salmonella enterica*, for example, H_2_O_2_ induces genes of the methylglyoxal pathway, which acts as a sink for glutathione (GSH) to enable the detoxification of harmful aldehydes produced during oxidative stress while enabling glucose utilization and aerobic respiration (71). MGO reacts with GSH to form an S-lactoylglutathione conjugate that is converted to lactate, fueling energetic and redox needs in cells experiencing oxidative stress (Fig. 2). The MGO pathway also promotes high glucose uptake and its utilization when the TCA cycle is repressed under oxidative stress, helping to recycle phosphate from accumulating toxic phosphosugars (92–94). Thus, MGO represents an overflow pathway with versatile functions, including enhancing stress resistance while enabling metabolic reconfiguration. In summary, despite their seeming energetic inefficiencies, overflow pathways can fine-tune stationary-phase physiology and modulate homeostasis by controlling levels of incompletely oxidized metabolites that possess regulatory functions. As a covalent protein modification, the potential of glycation by MGO to regulate transcription factors or enzyme activities merits further investigation.

## FUTILE CYCLES: NOT SO FUTILE

**Fig 3:**
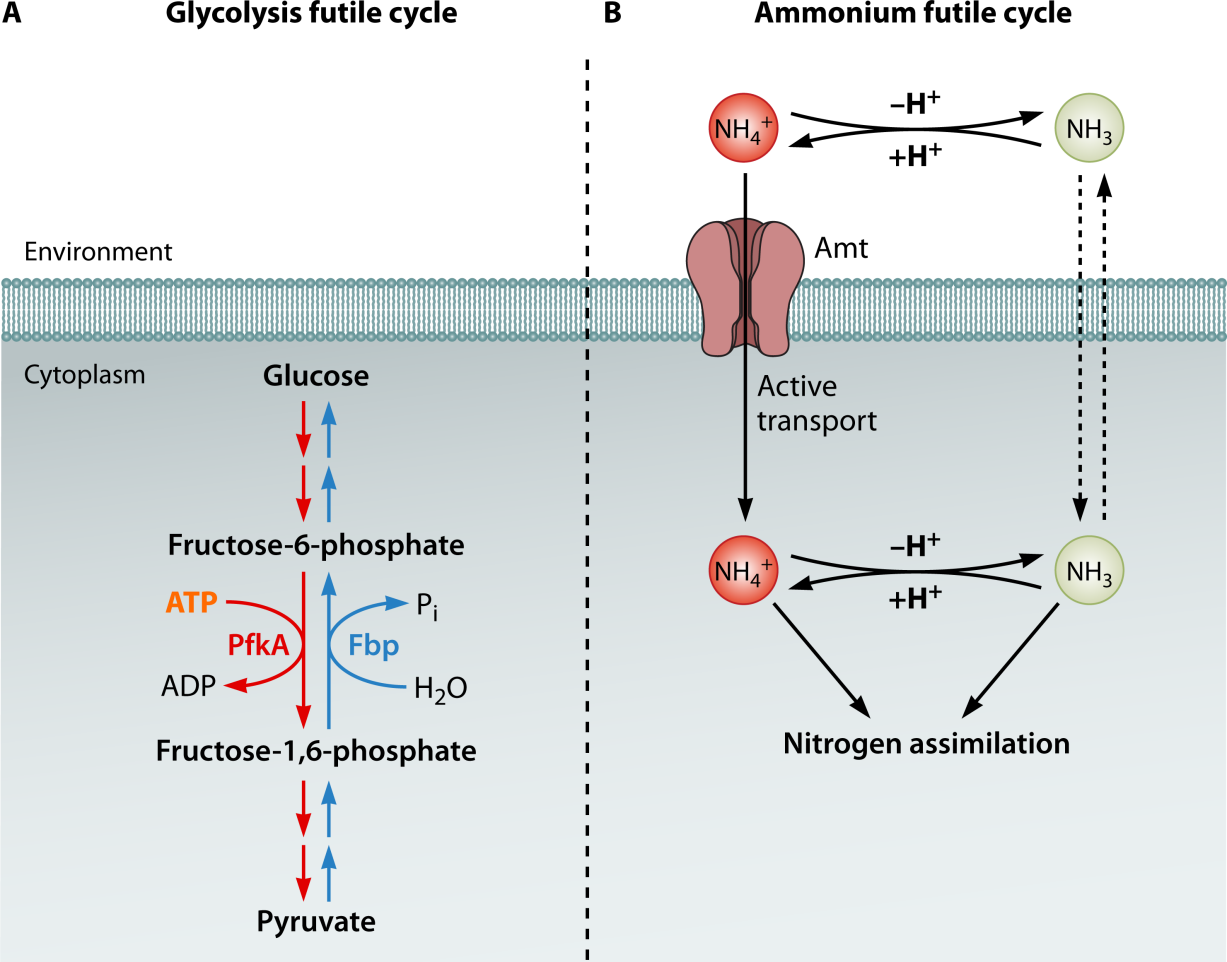
Energy-dependent futile cycles. (**A**) When glycolysis and gluconeogenesis operate simultaneously, the continuous interconversion between fructose-6-phosphate and fructose-1,6-bisphosphate can occur, resulting in the net loss of ATP. The enzyme phosphofructokinase (PfkA) converts fructose-6-phosphate to fructose-1,6-bisphosphate, using ATP, while fructose-1,6-bisphosphatase (Fbp) reverses the reaction and hydrolyzes fructose-1,6-bisphosphate back to fructose-6-phosphate, releasing inorganic phosphate. (**B**) Concomitant active uptake and passive transport to the environment of a nutrient or substrate can constitute a futile cycle. Ammonium (NH_4_^+^) transported into the cell via an energy-dependent ammonium transporter (Amt) can be deprotonated to ammonia (NH_3_). The exact mechanism behind energy-dependent transport via Amt is unclear. A high internal concentration of NH_4_^+^/NH_3_ can cause NH_3_ to diffuse back into the environment. In the environment, ammonia may accept a hydrogen ion (H^+^) to convert to ammonium, which can be taken up by active transport again.

Futile cycles represent another energy-spilling mechanism with increasingly recognized signaling properties. Futile cycles, also termed “substrate cycles,” occur when opposing metabolic pathways operate simultaneously, resulting in the concurrent synthesis and degradation of a metabolite with an associated net loss of energy (95). In some cases, regulatory mechanisms mitigate this waste through post-translational modifications of enzymes that control the activity of counteracting enzymes. A classic example is the interconversion of fructose-6-phosphate and fructose-1,6-bisphosphate in the central carbon metabolism in *E. coli*. The glycolytic step of the phosphorylation of fructose-6-phosphate is ATP dependent and is catalyzed by two different phosphofructokinases, Pfk-1 and Pfk-2. The reverse, gluconeogenic reaction, is catalyzed by fructose-1,6-bisphosphatase. When both glycolytic and gluconeogenic enzymes are active, a futile cycle of ATP hydrolysis occurs (Fig. 3A) (96, 97). Allosteric inhibition by MgATP of dimeric Pfk-2 leads to tetramerization and inhibition of enzyme activity, while increasing fructose-6-P levels counters this inhibitory effect, thus coordinating hexose sugar availability with glycolytic activity (51, 98).

Despite regulatory frameworks to reduce wasteful biochemical processes, various forms of futile cycles have been characterized across microbial systems. In lactose- and leucine-limited cultures of *Streptococcus cremoris*, the joint action of phosphofructokinase and fructose-1,6-bisphosphatase leading to ATP dissipation has been observed (99). In addition to simultaneous synthesis and degradation, concomitant import and export of substrates can also constitute a futile cycle since the substrate’s transport is energy dependent. Ammonium futile cycling, for example, has been demonstrated in some nitrogen-fixing bacteria, such as *Klebsiella pneumonia* (100, 101). Ammonia (NH_3_) readily diffuses through bacterial membranes, while ammonium ion (NH_4_^+^) uptake requires energy-dependent transport. Under certain growth conditions, newly produced ammonia can leak out of the cell; NH_4_^+^ is formed in the culture medium and subsequently taken up by the ammonium transporter, forming a futile cycle with energetic costs for nitrogen assimilation (Fig. 3B) (30, 102). Some polysaccharide pathways known to provide energy under conditions of starvation also exhibited futile cycling. In the cellulolytic bacterium *Fibrobacter succinogenes*, present in the rumen of cattle, glycogen is simultaneously stored and degraded during growth arrest (103), raising questions about the preconceived notion of glycogen as simply an energy-storing complex. Our own observations of stationary-phase *E. coli* suggest a potential role for glycogen futile cycling in modulating Cra signaling and as an overflow pathway for central carbon metabolism (data not shown).

Various metabolic and homeostatic functions have been ascribed to futile cycles, including energy dissipation during energy excess, control of metabolic sensitivity, and adaptive thermogenesis (37). The heat generated by futile cycles may provide a competitive advantage, as elevating local temperature can potentially alter growth rates (104) and may act as a defensive “fever” against bacteriophage infections (105). By acting as sinks for ATP or substrates, futile cycles may increase the metabolic sensitivity of pathways to substrate perturbations. Sensitivity in metabolic regulation is a measure of the relationship between the change in enzyme activity and the change in the concentration of the enzyme’s regulator. In a futile or substrate cycle, when the forward and reverse reactions are active, there is relatively little net generation of either reactant or product, and a small shift in the concentration of an allosteric regulator can drastically alter enzyme activity (36, 106).

Recent findings indicate that the ability of futile cycles to regulate the flux of central carbon metabolism implicates them as metabolic adaptation strategies to cope with variabilities in nutrient types. In fluctuating environments, the need to synthesize metabolic enzymes for different carbon sources, such as transitioning between glycolytic and gluconeogenic substrates, can increase the lag time prior to growth (107). Glycolysis and gluconeogenesis both include irreversible metabolic reactions that are regulated to mitigate futile cycling and enforce directional flux in central carbon metabolism. While simultaneous enzyme activity for these opposing pathways would expend energy and cause the circular conversion of metabolites in irreversible reactions, maintaining flux in both directions could accelerate the shift between glycolytic and gluconeogenic substrates (108). *In silico* modeling of *E. coli* has demonstrated such a trade-off, since increasing the abundance of both gluconeogenesis and glycolysis enzymes prior to the shift from one substrate source to another increases futile cycle activity yet decreases lag times (72). In another experiment subjecting cells to a regimen of alternating feast-famine conditions, *E. coli* was observed to use more glucose for respiration than biomass production, implying excess ATP was used in energy-spilling reactions. The authors speculated that futile cycles active during feast-famine conditions may underlie this ATP-spilling and could be functioning as a metabolic strategy to rapidly switch the direction of fluxes (109). These findings provide insight into the selective pressures in favor of futile cycles in dynamic environments, where they can confer metabolic versatility and responsiveness at the cost of ATP expenditure.

In addition to regulating metabolic responses, studies on the impact of futile cycling on global metabolism and transcription regulation reveal the capacity of futile cycles to modulate transcriptional regulation and thus alter system-wide metabolism (73, 95). In *E. coli*, promoting ATP or NADH wasting via plasmid-based overexpression of the soluble ATPase (*atpAGD*) or the NADH oxidase (*nox*), respectively, creates futile cycles for each cofactor and results in significant physiological and regulatory changes (73). Strains harboring either plasmid exhibit reduced growth rate and biomass yield. Notably, the artificial ATP or NADH sinks lead to increased glucose uptake and glycolytic rate, increased TCA cycle flux, and a major redistribution of metabolites and fluxes of central carbon metabolism. Perturbations in ATP and NADH levels also result in transcriptional changes that restore energy and redox homeostasis, respectively, including proton translocation mechanisms via the respiratory chain or activating enzymatic reactions that produce NADH. Metabolic reprogramming in these strains was triggered by the induction of global transcription factors such as ArcA, Fnr, IHF, Fur, and CRP, which control a wide spectrum of genes encoding metabolic pathways and functions, including aerobic metabolism, amino acid metabolism, and iron metabolism (73). In addition to the control exerted on metabolic regulation, futile cycles also play roles in stress responses. For example, promoting ATP futile cycling by overexpressing the soluble subunits of ATP synthase caused increased sensitivity to H_2_O_2_ and increased endogenous H_2_O_2_ production, demonstrating a relationship between futile cycling and oxidative stress (110). Thus, in response to futile cycles, metabolic and transcriptional changes restore energy homeostasis and redox ratios, leading to significant effects on cellular behavior and survival.

**Fig 4:**
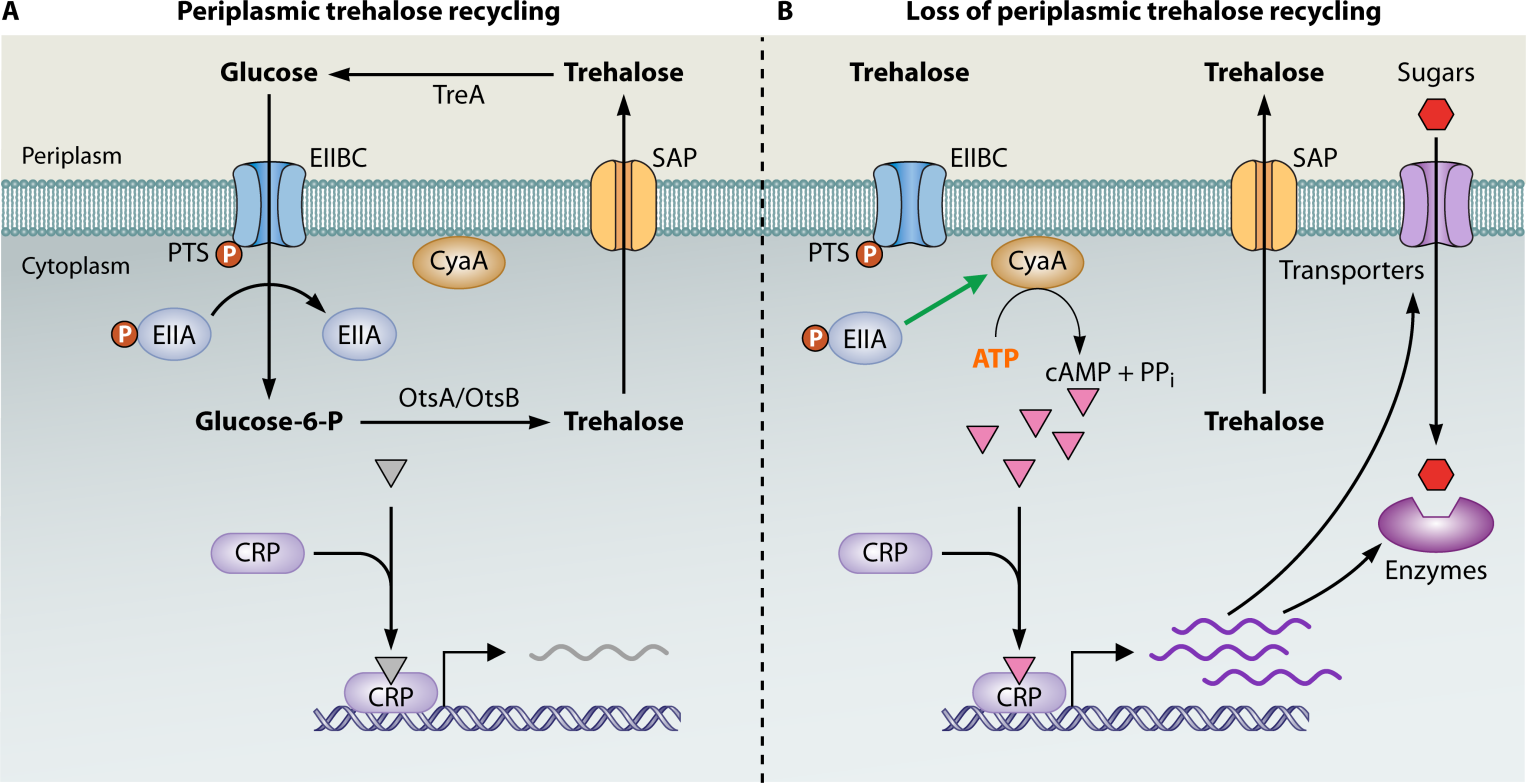
Trehalose futile cycling facilitates metabolic fine-tuning during stationary phase. (**A**) During stationary phase in *E. coli*, RpoS-induced expression of the biosynthetic genes *otsA* and *otsB* leads to the synthesis and accumulation of intracellular trehalose, which is then transported into the periplasm via stretch-activated porins (SAPs). Once in the periplasm, trehalose is hydrolyzed by TreA back into glucose. Flux of this liberated glucose through the PTS sugar transporter EIIC leads to the dephosphorylation of EIIA and EIIB, the phosphorylation state of which is key for modulating adenylate cyclase (AC) activity. Phosphorylated glucose can be converted back into trehalose or enter other metabolic pathways. (**B**) Deletion mutation of the periplasmic trehalase *treA* results in the accumulation of trehalose in the periplasm and reduced glucose flux through the PTS, leading to the phosphorylated EIIA-mediated activation of AC, which converts ATP into cAMP. Higher cAMP levels generate increased activation of the cAMP receptor protein (CRP), upregulating the expression of high- and moderate-affinity transporters for carbon substrates, as well as enzymes for alternative carbon metabolism (34).

Studies in our lab investigating the role of the metabolite trehalose, an endogenously synthesized disaccharide with stress protection properties, during feast-famine conditions identified a regulatory role for a novel futile cycle in *E. coli* (34) (Fig. 4). Trehalose has metabolic roles in various organisms, but it has been recognized solely as an osmoprotectant in *E. coli* (111). However, disrupting periplasmic trehalose degradation in *E. coli* induces unexpected metabolic reprogramming and behavioral changes. During the stationary phase, trehalose excreted into the cytoplasm is transported back into the periplasm via stretch-activated porins. Once in the periplasm, trehalose is hydrolyzed by the enzyme TreA into glucose (112). The flux of this liberated glucose through the PTS sugar transporter EIIC leads to the dephosphorylation of EIIA and EIIB, a phosphorylation state important for modulating adenylate cyclase activity and producing cAMP necessary to activate the transcription factor CRP (113). Glucose transported into the cytoplasm, a phosphorylation-dependent step, can be converted back into trehalose, which is transported back into the periplasm, repeating the cycle. Disrupting this trehalose futile cycle by knocking out the *treA* gene resulted in trehalose loss to the environment and elevated ATP levels, yet increased nutrient scavenging and elevated cAMP-CRP activity. Furthermore, a mutant strain lacking TreA possessed a significant competitive advantage against wild-type cells in mixed culture studies. The coupling of this futile cycle (an ATP-dependent pathway involving the simultaneous synthesis and degradation of trehalose) with the PTS (which functions as a flux sensor for glucose) may enable the cell to monitor and respond to its own carbon levels (34). In other words, a futile cycle active under stress can serve as a critical modulator of carbon starvation responses during the stationary phase. Thus, via their impact on system-wide metabolic fluxes and global transcriptional signaling, futile cycles can influence metabolic homeostasis and thereby act as a determining factor for survival in complex, dynamic environments.

## BROADER IMPACTS: UTILITY EMERGING FROM FUTILITY

Futile cycling and overflow metabolism are increasingly recognized as key modulators of physiological programming and system-level metabolic flux. The two types of energy-spilling reactions can be interconnected. Futile cycling resulting in higher metabolic rates can trigger overflow metabolism at the expense of growth yield (95). Alternatively, overflow metabolites, such as acetate, can be catabolized and synthesized concurrently, manifesting in a futile cycle (114). The influence of these energy-spilling reactions over microbial signaling and stress responses harbors both positive and negative ramifications for intraspecies and host-microbe interactions. Fermentation products generated through overflow metabolism can function as intracellular metabolites to mediate cross-feeding interactions. Nitrogen overflow by the yeast *Saccharomyces* cerevisiae, for example, supplies amino acids for *Lactococcus lactis*, enabling a mutualism to emerge in a lactose medium (115). Exchanges of overflow metabolites in the gut microbiota may be an important driver of community stability and resilience, contributing to species fitness and host health (116). In contrast to this intraspecies collaboration, toxic overflow products can function antagonistically and promote resistance to invasion. For example, lactic acid generated by vaginal *Lactobacilli* can function as a potent microbicide that limits infection by acid-sensitive pathogens (117, 118). In addition, overflow products can function as important signaling molecules and modulators of host fitness. Specifically, bacteria-derived MGO can modulate the longevity of host *Caenorhabditis elegans* through the regulation of a host signaling pathway (119, 120). Finally, our work and that of others suggest that futile cycles can shape intraspecies dynamics, where altered transcriptional signaling and metabolic activity can modify fitness and shift the balance in competitive and stressful environments (34, 109).

Energy-spilling pathways are also novel targets for optimization in metabolic engineering and synthetic biology. The formation of overflow metabolites, especially acetate, by *E. coli* and other microbes hampers large-scale fed-batch processes and can have adverse effects on target product formation and cell growth (121). Mutations that channel flux away from the acetate precursors pyruvate and acetyl-CoA and into the TCA cycle reduced acetate synthesis and enhanced cell robustness (122). In contrast, an industrial strain of *Bacillus subtilis* was engineered with a heterologous metabolic pathway to increase the catabolism of excreted acetate. Enzymes of the glyoxylate shunt from *Bacillus licheniformis* can be expressed in *B. subtilis*, allowing this strain to utilize excreted acetate for improved growth behavior (123). Similar metabolic engineering interventions that regulate and minimize production of overflow metabolites are vital to optimize microbial cell factories and increase the production of target compounds.

Exploiting futile cycles through engineering also offers another means to optimize substrate-product yields by stimulating ATP wasting to improve the properties of cell factories (124). For example, promoting ATP hydrolysis by introducing a futile cycle shifts net flux from biomass to product synthesis, resulting in increased anaerobic product yields. ATP wasting via the pyruvate kinase/PEP synthase futile cycle, for instance, increases anaerobic lactate production in *E. coli*. (125). Therefore, elucidating energy-utilizing processes can reveal strategies to manipulate microbial metabolism for a wide spectrum of biotechnology applications and microbial production.

**Fig 5:**
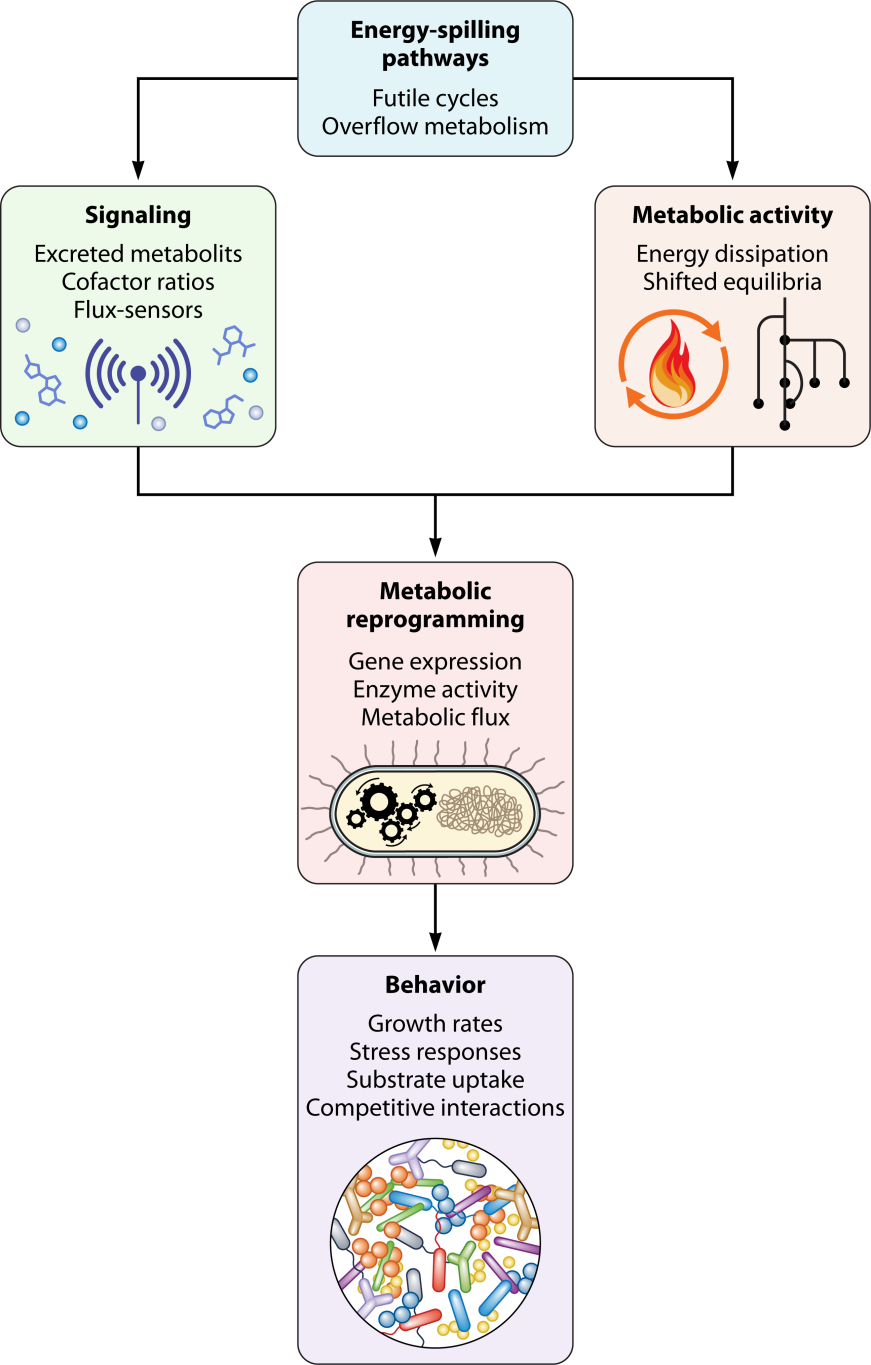
Energy-spilling pathways and their implications for microbial systems. Experimental evidence supports a role for energy-spilling pathways (futile cycles and overflow metabolisms) in altering microbial metabolism and behavior. These pathways can produce metabolites, modify cofactor ratios (e.g., NAD/NADH), and stimulate flux sensors that are the inputs for intracellular metabolic signaling. By dissipating ATP and shifting metabolic equilibria, futile cycles and overflow metabolism can fine-tune metabolic pathway fluxes and alter the cell’s energy state, thereby affecting metabolic activity. These intracellular processes can activate metabolic reprogramming at the levels of gene expression, enzyme activity, and pathway flux. This reprogramming can enable cells to switch to different metabolic strategies in response to challenging environments. These adjusted cellular adaptations and behaviors, such as increased substrate uptake or modulated stress responses, ultimately impact survival and shape environmental responses and competitive interactions.

Understanding energy-spilling reactions as molecular mechanisms for metabolic reprogramming enhances our fundamental understanding of adaptations to nutrient- or energy-limited environments (Fig. 5). This paradigm also prompts a reevaluation of the parameters of starvation states, energy limitation, and quiescent states for microbial systems. Specifically, the presence of uneconomical pathways, such as futile cycles, during growth stasis implies that energy may in fact be sufficient for survival even when cell growth is not observed. Instead of rationing limited resources exclusively for essential pathways, cells can respond by activating seemingly wasteful pathways and transition to a homeostatic state of reduced but sustained metabolism prior to depletion of extracellular resources. Thus, microbes may not be starving *per se* but rather persisting in a new homeostatic state. Experimental systems that recapitulate starvation physiology model microbial life under such demanding growth constraints but not necessarily energy deprivation.

## CONCLUSION

Historically, microbial metabolic systems were thought to be organized and regulated to minimize energy loss, maximize fuel-harnessing processes, and streamline complex biochemical pathways into efficient metabolic networks. A growing body of experimental evidence challenges these assumptions. The prevalence and utility of energy-spilling mechanisms in microorganisms contrast with long-held views of maximal energy efficiency in metabolic pathways (Table 1). However, energy spilling may be a misnomer since, despite the superfluous energy expenditure, these pathways help optimize overall metabolism. An emerging conceptual framework suggests that metabolism not only supplies the cell with energy and biochemical building blocks but also operates as an intricate signaling network interlocking metabolic cues with regulatory mechanisms. A metabolite’s value should be measured not only simply by its efficiency in transforming extracellular substrates directly into energy and biomass but also by its capacity to mediate metabolic reprogramming. This regulatory framework offers an opportunity to explore overflow metabolism and futile cycles as costly systems that facilitate intracellular signaling through interactions with enzymes or transcription factors and alter cofactor ratios that serve as the intracellular gauges of energy and redox homeostasis.

Furthermore, energy-spilling reactions under “starvation” conditions not only reaffirm that non-growing cells are metabolically active but also challenge the paradigm of cells subsisting strictly via maintenance metabolism in the absence of growth. The observation of futile cycles and overflow metabolism in stationary-phase cells demands a reevaluation of our understanding of starvation in microbial systems. Energy-spilling reactions may represent a surplus of energy redirected from biomass production to overflow metabolite synthesis and ATP-expending reactions, signifying a new homeostatic state rich with diverse and underexplored metabolisms. The so-called energy-wasting pathways enrich our understanding of how metabolism biochemically entangles the cell with its environment, transposing external and internal stimuli into the triggers for cellular decisions. Such multipurpose and essential utilities merit a revision of the term wasteful pathways, an incongruity that ignores an emerging theory of “utility from apparent futility (37).”
